# Maintenance of Transcription-Translation Coupling by Elongation Factor P

**DOI:** 10.1128/mBio.01373-16

**Published:** 2016-09-13

**Authors:** Sara Elgamal, Irina Artsimovitch, Michael Ibba

**Affiliations:** Department of Microbiology, The Ohio State University, Columbus, Ohio, USA

## Abstract

Under conditions of tight coupling between translation and transcription, the ribosome enables synthesis of full-length mRNAs by preventing both formation of intrinsic terminator hairpins and loading of the transcription termination factor Rho. While previous studies have focused on transcription factors, we investigated the role of *Escherichia coli* elongation factor P (EF-P), an elongation factor required for efficient translation of mRNAs containing consecutive proline codons, in maintaining coupled translation and transcription. In the absence of EF-P, the presence of Rho utilization (*rut*) sites led to an ~30-fold decrease in translation of polyproline-encoding mRNAs. Coexpression of the Rho inhibitor Psu fully restored translation. EF-P was also shown to inhibit premature termination during synthesis and translation of mRNAs encoding intrinsic terminators. The effects of EF-P loss on expression of polyproline mRNAs were augmented by a substitution in RNA polymerase that accelerates transcription. Analyses of previously reported ribosome profiling and global proteomic data identified several candidate gene clusters where EF-P could act to prevent premature transcription termination. *In vivo* probing allowed detection of some predicted premature termination products in the absence of EF-P. Our findings support a model in which EF-P maintains coupling of translation and transcription by decreasing ribosome stalling at polyproline motifs. Other regulators that facilitate ribosome translocation through roadblocks to prevent premature transcription termination upon uncoupling remain to be identified.

## IMPORTANCE

Bacterial mRNA and protein syntheses are often tightly coupled, with ribosomes binding newly synthesized Shine-Dalgarno sequences and then translating nascent mRNAs as they emerge from RNA polymerase. While previous studies have mainly focused on the roles of transcription factors, here we investigated whether translation factors can also play a role in maintaining coupling and preventing premature transcription termination. Using the polyproline synthesis enhancer elongation factor P, we found that rapid translation through potential stalling motifs is required to provide efficient coupling between ribosomes and RNA polymerase. These findings show that translation enhancers can play an important role in gene expression by preventing premature termination of transcription.

## INTRODUCTION

In *Bacteria*, mRNA translation can initiate as soon as the ribosome-binding site (RBS) emerges from RNA polymerase (RNAP), and the translating ribosome closely follows the elongating RNAP during the first round of translation (1). Coupling between transcription and translation is maintained in part by ribosomal protein S10, which may interact with RNAP (1) and NusG (2, 3). Interplay between transcription and translation plays a central role in controlling gene expression; for example, trailing ribosomes can prevent formation of nascent terminator hairpins (4) or occlude binding of the transcription termination factor Rho (5). Close coupling of transcription and translation also ensures continual synthesis of cellular mRNAs—the trailing ribosome blocks RNAP backtracking that leads to arrest, and in so doing inhibits premature transcription termination (1, 6, 7). Conversely, when translation slows and is uncoupled from transcription, nascent mRNAs become susceptible to Rho-mediated release (transcriptional polarity [8]). Rho-dependent terminators are found in leader regions, within coding sequences, and at the ends of transcriptional units (9) and account for at least 20% of the identified transcription terminators in *Escherichia coli* (10).

**FIG 1:**
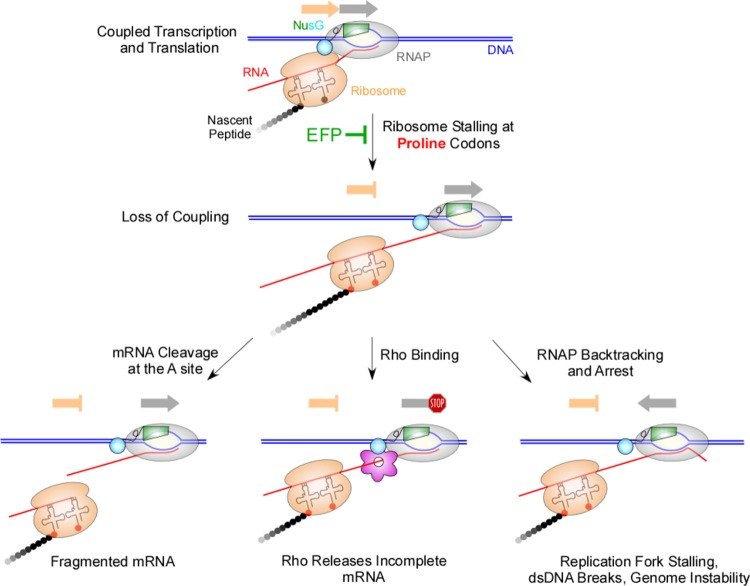
Consequences of ribosome stalling on the fate of the nascent mRNA. (Top) During the first round of translation, RNAP and ribosomes move in tandem (arrows). In the absence of EF-P, ribosomes may stall on mRNAs with consecutive proline codons, whereas RNAP keeps moving. (Bottom) Unresolved translational arrest can lead to mRNA cleavage at the A site (left), Rho-mediated transcription termination (center), or transcriptional arrest upon RNAP backtracking (right). dsDNA, double-stranded DNA.

Variations in translation elongation rates have been implicated in gene regulation (11), mRNA decay (12, 13), codon bias (14), and protein folding (15). The mechanism of addition is the same for all amino acids during protein synthesis; however, the speed of peptidyl transfer is not uniform, and ribosomes elongate at different rates (16). Among all genetic code amino acids, proline displays by far the lowest rate of peptidyl transfer on the ribosome (17, 18). Proline is the only natural cyclic amino acid, and its pyrrolidine ring imposes structural constraints on the positioning of the amino acid in the peptidyl transferase center, resulting in slow peptide bond formation and occasional ribosome stalling (19). To overcome this potential bottleneck, robust synthesis of proteins with consecutive prolines requires elongation factor P (EF-P) (20–24). EF-P and its eukaryotic homolog eIF5A prevent ribosome pausing and stalling during polyproline synthesis by increasing the rate of peptide bond formation entropically via positioning and stabilization of peptidyl-Pro-tRNA^Pro^ (20, 25–27). Ribosome stalling caused by certain peptides may lead to mRNA cleavage around the stop codon (28, 29) or at sense codons when translation elongation is prevented (30). Stalling translation elongation can also have deleterious effects on transcription, including premature termination and RNAP backtracking (Fig. 1). The backtracked RNAP is incapable of nucleotide addition but is stably bound to the DNA, blocking the progression of the replication fork and introducing single-strand and double-strand DNA breaks (DSBs), leading to increased mutation rates and genome instability (31).

While loss of coupling during translation of a defective mRNA represents an important quality control surveillance mechanism, transient ribosome stalling on functional messages must be minimized to ensure uninterrupted mRNA synthesis. In this work, we investigated the impact of the translation anti-pausing factor EF-P on the coupling of transcription to translation across an EF-P-dependent motif. Utilizing a reporter with a polyproline motif upstream of a termination site, it was shown that the absence of EF-P promoted uncoupling, thereby allowing premature RNA release. These findings show that EF-P plays an important role in gene expression by minimizing reductions in translation elongation rates that would otherwise lead to uncoupling from RNAP.

## RESULTS

### Efficient polyproline synthesis decreases Rho-dependent transcription termination.

**FIG 2:**
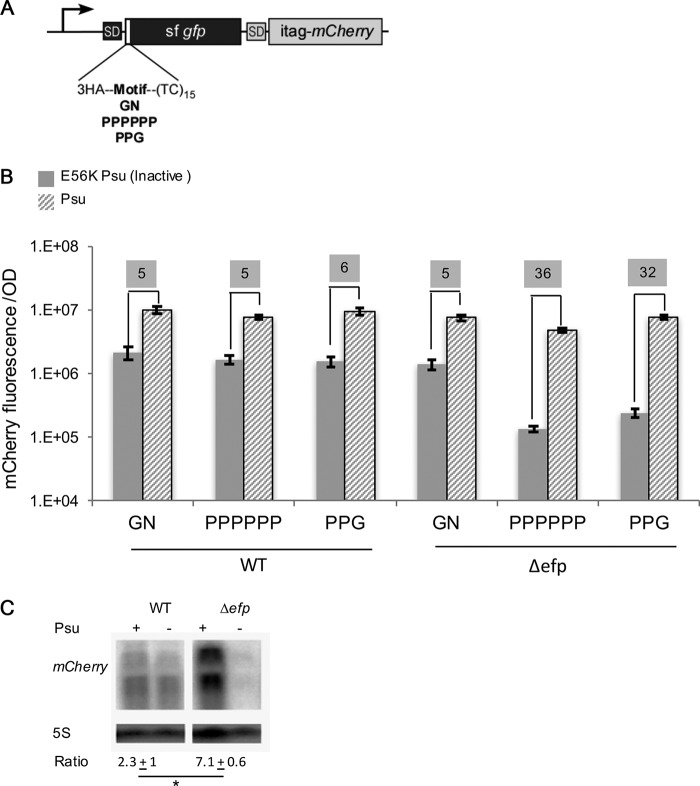
Stall motifs reduce reporter expression in a Rho-dependent manner in the absence of EF-P. (A) pBAD30HATC (*rut*) reporter with three different inserts, GN, PPPPPP, and PPG, followed by a (TC)_15_ *rut* element. SD, Shine-Dalgarno sequence. (B) mCherry fluorescence/OD for WT and *Δefp* strains harboring both the *rut* reporter and a compatible plasmid producing either active or inactive E56K mutant Psu; an empty vector lacking Psu showed the same results as inactive Psu (data not shown). The values shown on a gray background are the fold changes in mCherry fluorescence with coexpression of Psu. The means for at least three biological replicates are shown, and error bars indicate 1 standard deviation. (C) Representative Northern blot for *gfp*-*mCherry* with PPPPPP insert for both WT and *Δefp* strains. The mean ± SD are shown (*n* = 2). The increase in mCherry in the presence of PSU was significantly different (*P* < 0.05) as determined by unpaired Student’s *t* test as indicated by the asterisk.

To investigate whether ribosomal stalling in a *Δefp E. coli* strain can uncouple translation from transcription, a reporter was constructed with a transcriptional fusion of *sfgfp* (superfolder green fluorescent protein [sfGFP]) and *mCherry*, each with a Shine-Dalgarno sequence (Fig. 2A). GN, PPPPPP, and PPG motifs were inserted at the beginning of *sfgfp*; PPPPPP and PPG motifs induce ribosome stalling that can be alleviated by EF-P (22). Rho is an ATP-dependent RNA translocase and helicase that binds to a Rho utilization (*rut*) site on the nascent mRNA, translocates along the RNA toward the RNAP, and finally triggers the dissociation of the transcription elongation complex (32). In the classic model, Rho tracks along the RNA until it reaches the elongating RNAP and pulls out the RNA from the enzyme by means of its helicase activity (33). In an “allosteric” model, Rho associates with RNAP throughout transcription and, following the capture of *rut*, pulls the nascent RNA through, progressively shortening it and accumulating topological stress that inactivates the elongation complex by destabilizing the RNA-DNA hybrid (34). In either model, a closely coupled ribosome would inhibit Rho-mediated termination.

To assess uncoupling, a (TC)_15_ *rut* site was placed downstream of the peptide motif. This *rut* site was previously shown to lead to Rho-dependent termination in the absence of protein synthesis (32). To explore the extent to which the observed effect is attributable to Rho, which is essential in *E. coli* (35), Psu, a bacteriophage P4 protein (36) that inhibits Rho with high efficiency and specificity (37, 38), was expressed from a compatible plasmid. If ribosome stalling at PPX motifs uncouples transcription and translation, the *rut* site would be expected to decrease synthesis of superfolder GFP (sfGFP) and mCherry in Δ*efp*, but not wild-type (WT), *E. coli* strains. In the absence of Psu or in the presence of the inactive E56K mutant Psu (which lacks the ability to bind Rho [38, 39]), a >30-fold decrease in mCherry fluorescence was observed from reporters encoding PPX motifs in the Δ*efp* strain, compared to only a 5-fold decrease in the WT strain(Fig. 2B, shown on gray background); similar changes in GFP fluorescence were observed (see Fig. S1A in the supplemental material). In contrast, the GN reporter showed similar levels of expression in the presence and absence of EF-P. Notably, in the WT strain, all reporters produced the fluorescent proteins similarly and showed ~5-fold improved mCherry fluorescence when Rho was inhibited by Psu (Fig. 2B, shown on gray background). This apparent Rho-mediated polarity may indicate the background, stalling-independent, level of uncoupling, for example due to ribosomes that fail to engage mRNAs early during their synthesis.

To confirm that the observed effects on fluorescent protein production were due to changes in mRNA abundance, mCherry transcript levels were quantified (Fig. 2C). Two hybridization products were observed, one of higher weight representing the full-length *gfp-mCherry* transcript (~2,000 nucleotides [nt]) and a second transcript (~1,200 nt). The smaller product is consistent with termination at a stem-loop structure predicted by ARNold (40, 41), (AGUGACUAAAGGCGGCCCGCUGCCUUUUGCGUGGGA; free energy of −8.40 kcal/mol [42]). Transcript levels for *gfp-mCherry* produced from the PPPPPP reporter in the WT strain were ~2-fold higher in the presence of Psu, consistent with residual polarity noted above. In the *Δefp* mutant, the transcript level for *gfp-mCherry* was ~7-fold higher when Rho was inhibited by Psu production (Fig. 2C). Together, these observations support the proposed role of EF-P in preventing uncoupling.

### EF-P effect is potentiated by accelerating transcription.

Coupling of transcription and translation implies that RNAP and the ribosome move at identical rates (43), yet the speed of either machinery can be modulated by nucleic acid signals and accessory factors in the cell. The above results indicate that EF-P can maintain coupling by alleviating ribosome stalling at polyproline motifs. However, coupling could also be broken if RNAP is moving too fast, for example, upon modification by a transcription antitermination factor (44). We hypothesized that the effect of *efp* deletion will be exacerbated by a mutation in RNAP that increases the rate of RNA synthesis. We compared the effects of EF-P on expression of mCherry in strains containing the WT *rpoB* gene and a “fast” *rpoB2* allele, which encodes the βH526Y substitution in *E. coli rpoB*. RpoB2 enzyme has been shown to decrease termination at some sites (45) and increase transcript elongation rate *in vivo* and *in vitro* (39, 46, 47).

**FIG 3:**
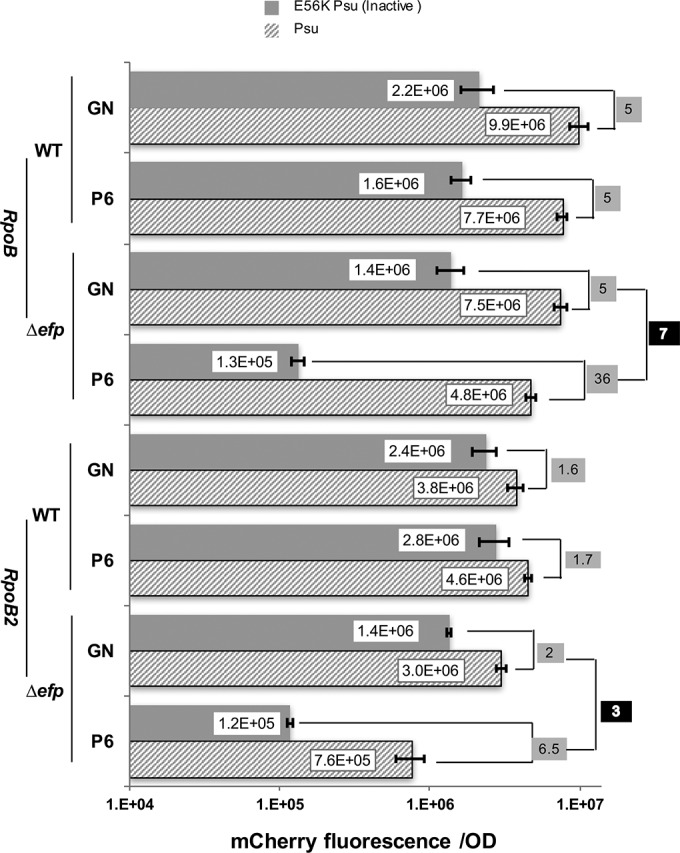
Fast RNAP facilitates uncoupling. mCherry fluorescence/OD compared in *rpoB* (data from Fig. 1B) and *rpoB2* strains. The values shown on a gray background are the fold changes in mCherry fluorescence with coexpression of Psu. The values shown on a black background are the ratio between PPPPPP (P6) to GN insert. The means for at least three biological replicates are shown on the graph, and error bars indicate 1 standard deviation.

In the WT strain when the GN motif was replaced with PPPPPP with active Rho, mCherry production decreased by ~27% in the *rpoB*^+^ RNAP background (Fig. 3, 2.2E+06 to 1.6E+06) and increased by ~8% in the *rpoB2* strain (2.4E+06 to 2.8E+06). In the presence of active Psu with the PPPPPP motif, expression decreased by ~20% in the *rpoB* RNAP background (9.9E+06 to 7.7E+06) and increased by ~20% in the *rpoB2* strain (3.8E+06 to 4.6E+06). The differences between the *rpoB* and *rpoB2* strains suggest that an engineered PPPPPP motif serves as a weak pause/arrest signal with the *rpoB* RNAP enzyme but not with *rpoB2* RNAP, which would be expected to bypass pause signals efficiently (46). Also inhibition of Rho by Psu led to smaller increases in expression by the *rpoB2* RNAP (~1.6-fold) compared to the *rpoB* RNAP (5-fold [highlighted in grey in Fig. 3]). These observations are consistent with a faster transcription elongation rate in *rpoB2* (39), which makes it more resilient to Rho (48, 49) and thus less susceptible to inhibition by Psu.

In contrast, in the *Δefp* strain insertion of the PPPPPP motif led to a large decrease in mCherry production, particularly when Rho was active (more than 10-fold with either RNAP). When Rho was inhibited by Psu, the observed PPPPPP effect was greater with the B2 RNAP (4-fold, 3E+06 to 7.6E+05) than with the *rpoB* RNAP (1.5-fold, 7.5E+06 to 4.8E+06), consistent with the idea that a fast RNAP would exacerbate uncoupling triggered by ribosome stalling. With both reporters, expression of Psu stimulated mCherry production by either RNAP, but to a different extent. A comparable increase was also detected in mRNA levels (see Fig. S1B in the supplemental material). In the case of the *rpoB* RNAP, the level of mCherry production was increased 7-fold but increased only 3-fold with the *rpoB2* enzyme (Fig. 3, highlighted in black). The greater defect observed with the *rpoB2* RNAP reveals additive effects of reducing coupling by ribosome stalling (in the absence of EF-P) and RNAP acceleration (by H526Y substitution). Together, these results indicate that EF-P can compensate for defects in protein production under conditions when coupling between RNAP and the ribosome is broken by disparate changes in their rates.

### Efficient polyproline synthesis maintains transcription through intrinsic terminators.

**FIG 4:**
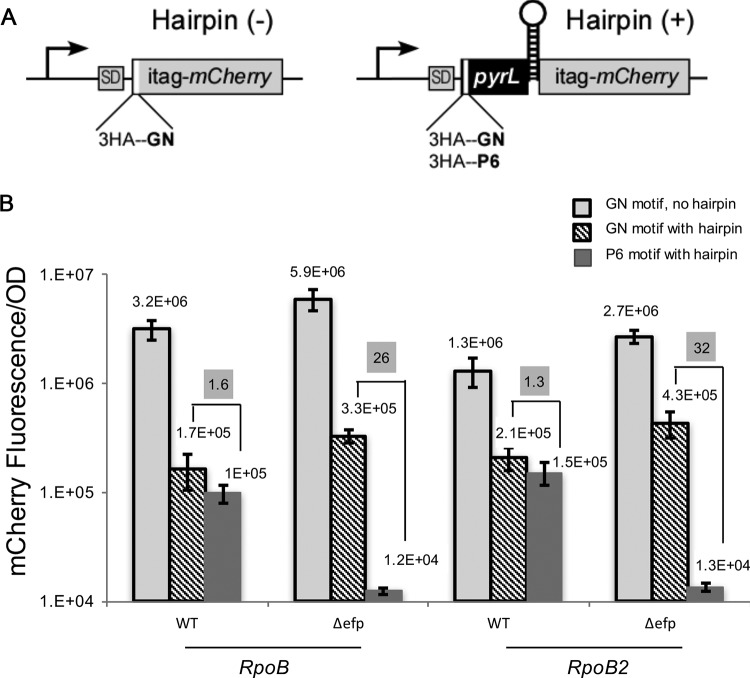
Reporter with an intrinsic terminator downstream of a polyproline motif. (A) pBAD30HAPYT plasmid with a PPPPPP or GN insert. In the reporter with a hairpin (+), the insert is followed by *pyrL*. The region before the insert is a three-hemagglutinin (3HA) tag. (B) mCherry (or *pyrL*-mCherry) fluorescence/OD for WT and *Δefp* strains harboring pBAD30HAPYT plasmid with a PPPPPP or GN insert. For each strain, two *rpoB* chromosomal backgrounds were tested, *rpoB* (WT) and *rpoB2.* The values shown on a gray background are the fold changes in mCherry fluorescence in the presence of hairpin. The means for at least three biological replicates are shown on the graph, and error bars indicate 1 standard deviation.

The above results establish the role of EF-P in preventing premature Rho-mediated termination. To investigate the effects of ribosome stalling on Rho-independent termination, a reporter was constructed in which a stall site (PPPPPP) was placed upstream of the intrinsic terminator hairpin from the *pyr* leader region (Fig. 4A), where an advancing ribosome has previously been shown to inhibit termination (50).

In the *rpoB* RNAP background with the GN motif, we observed that insertion of the *pyrL* hairpin terminator (GN motif with hairpin) reduced the mCherry fluorescence ~18-fold compared to when the hairpin was absent (GN motif with no hairpin) from either the WT or Δ*efp* strains (Fig. 4B, 3.2E+06/1.7E+05 and 5.9E+06/3.3E+05, respectively), consistent with the published results (4). Since close coupling would be expected to prevent the formation of the terminator hairpin, this result indicates that coupling did not occur on most *pyrL-*mCherry RNAs, consistent with the polarity observed with the *rut* reporter (Fig. 2). Further decreased coupling due to ribosome stalling would be expected to additionally increase termination. To investigate this, a PPPPPP motif was inserted upstream of the hairpin. In the WT, PPPPPP insertion caused less than a 2-fold decrease in mCherry fluorescence compared to a 26-fold decrease in the Δ*efp* strain (Fig. 4B, shown on gray background). A similar decrease was also detected in mRNA levels (see Fig. S1C in the supplemental material).

We next tested whether accelerating transcription in the *rpoB2* background would augment the effect of *efp* deletion. We note that the effects of accelerated transcription on the reporter production are complex: on one hand, RpoB2 substitution promotes the terminator hairpin formation via uncoupling, while on the other hand, RpoB2 directly inhibits termination. However, its functional interaction with EF-P could be deduced from the analysis of the EF-P-dependent reporters containing the PPPPPP motif. In the absence of the stalling motif (GN motif), the *pyrL* hairpin terminator reduced mCherry expression by B2 RNAP only 6-fold in both the WT and Δ*efp* strains (Fig. 4B, 1.3E+06/2.1E+05 and 2.7E+06/4.3E+05, respectively); similar *rpoB2* defects were observed at several intrinsic terminators *in vivo* (45). Addition of the PPPPPP motif decreased mCherry production 1.3-fold in the WT strain, but 32-fold in the Δ*efp* mutant background (Fig. 4B, shown on gray background), a modest but significant increase over the 26-fold effect observed in the *rpoB*^+^ background. These results support a model in which EF-P prevents uncoupling at polyproline motifs, thereby inhibiting premature RNA release at an intrinsic terminator.

### EF-P suppresses Rho-dependent transcription termination *in vivo.*

While the major known function of EF-P is to facilitate the robust synthesis of polyproline motifs, this may not provide a complete picture of the role of EF-P in gene expression. The proteomic data from stable isotope labeling with amino acids in cell culture (SILAC) experiments performed in both *Salmonella enterica* serovar Typhimurium (22) and *E. coli* (24) indicated that only a minority of proteins containing the PPX motif were downregulated in the *Δefp* strain. In *E. coli*, ~13% of proteins and ~21% of mRNAs displayed at least 2-fold differences between the WT and *Δefp* strains, as measured by SILAC (24) and transcriptome sequencing (RNA-seq) (23), respectively. In both data sets, PPX-containing genes are almost equally represented in the upregulated and downregulated genes, suggesting that EF-P may have effects on both transcription and translation, consistent with the data presented above. It is conceivable that ribosomes stalling at polyproline motifs could favor formation of either a terminator or antiterminator RNA structure, thus enhancing or decreasing termination efficiency, respectively.

In our analysis, we utilized artificial constructs in which strong Rho-dependent or intrinsic termination signals were placed between a PPPPPP motif and a reporter gene. While these model terminators enabled us to demonstrate a role for EF-P in transcription-translation coupling, understanding of the physiological effects of EF-P requires identification of its cellular targets. We sought to identify termination signals sensitive to EF-P *in vivo*. Bioinformatic prediction of Rho-dependent terminators is not feasible, since other than C richness, there are very few other conserved features (51), and the accessibility of RNA is a determining factor in Rho recruitment. Although a few selected strong Rho-dependent terminators have been studied extensively, less is known on a genome-wide scale. Recent work by Peters et al. (52) used two strand-specific, global RNA-profiling techniques to obtain high-resolution maps of Rho-dependent termination in WT *E. coli*, which would not directly target detection of any EF-P-dependent effects.

**FIG 5:**
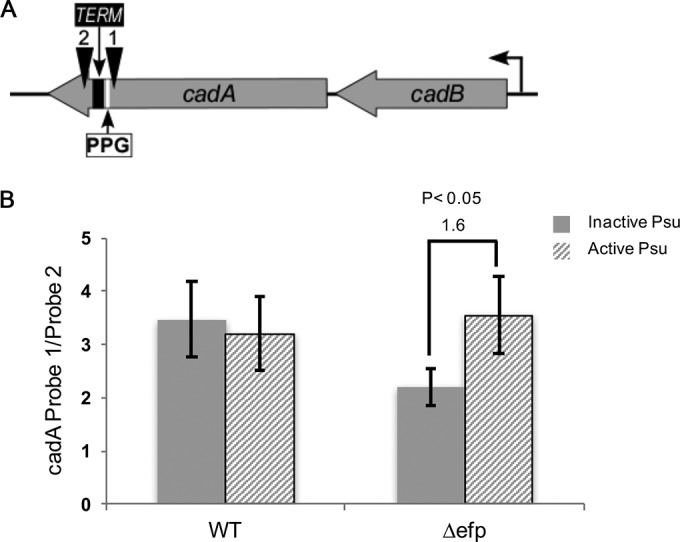
*In vivo* probing for *cadBA*. (A) Representation of the *cadBA* operon with the location of the PPG motif, a predicted terminator (TERM), and the probes (C1 and C2 probe) indicated. (B) Quantification of the dot blot for samples examined with probes C1 and C2. The means for at least three biological replicates are shown, and error bars indicate 1 standard deviation.

PPX-containing proteins downregulated in the Δ*efp* strain in both RNA-seq and SILAC data sets (Table 1) could represent EF-P targets. For regions downstream of the PPX motif, C/G ratios and secondary structure propensities were determined. There is an observed depletion of G residues from *rut* sites (51), which is believed to play an indirect role in Rho binding by making the RNA less likely to form strong secondary structures (10). Among several genes having C-rich regions predicted to be devoid of secondary structures, only *cadA* was shown to have a Rho-dependent terminator (52), which starts 40 bp before the stop codon of *cadA*. The PPG motif in *cadA* is 144 bp upstream of this *rut* site (Fig. 5A). To investigate the effects of PPG and EF-P on *cadA* termination, we utilized hybridization probes complementary to sequences before and after the PPG motif. The contribution of Rho-dependent termination was assessed by additionally probing for WT and *Δefp* strains expressing Psu. The dot blot (see Fig. S2A in the supplemental material) indicated no change in the before/after probe ratio for the WT whether Psu was expressed or not. This could be expected for the WT, since Rho cannot load onto a *rut* site when transcription and translation are coupled. In the *Δefp* strain, a 1.6-fold reduction in the before/after probe ratio was detected in the absence of Psu (Fig. 5B), consistent with the notion that ribosomal stalling at PPG motifs in the absence of EF-P can uncouple transcription and translation, thus allowing Rho termination. In contrast, analyses performed for *ygdH* and *hslU* did not reveal any differences in the *Δefp* strain (Fig. S3 and S4), consistent with the absence of Rho-dependent signals in these genes (52).

**TABLE 1:** Common genes identified from both SILAC (24) and RNA-seq (23) that are downregulated in a *Δefp* strain compared to WT *E. coli* and contain at least one PPX motif

Gene^a^	WT/Δ*efp* ratio		Polyproline motif	Within 200 bp after PPX	
	SILAC score^b^	RNA-seq^c^		Highest %C/%G ratio^d^	Highest initial Δ*G* (kcal/mol) for predicted RNA secondary structure^e^
*acnB*	2.73	2.77	PPA	62.5:0	−56.9
			PPG	62.5:12.5	−59.8
			PPT	62.5:0	−68.9

*cadA*	6.78	3.24	PPL	37.5:0	−54.8
			PPG	37.5:12.5	−55.6

*ygdH*	3.28	2.54	PPE	62.5:12.5	−51.2
			PPN	62.5:12.5	−59.1

*yghJ*	2.25	4.43	PPV	50:0	−62.8
			PPR	37.5:0	−66.8

*hslU*	2.07	2.63	PPA	62.5:12.5	−63.8
			PPG	37.5:12.5	−56.5

*mqo*	2.98	6.58	PPM	62.5:0	−47.6

*ybiU*	5.09	3.62	PPG	50:0	−70.1

a These genes have at least a twofold difference in the total RNA reads in the RNA-seq data set.

b SILAC data from reference 24.

c RNA-seq data from reference 23.

d C and G percentages were calculated within windows of 8 bp.

e Δ*G* prediction for RNA secondary structure by Mfold (74).

### Potential intrinsic termination sites.

An intrinsic terminator is an RNA signal composed of a GC-rich stem-loop structure followed by a U-rich region (51). For all the PPX-containing genes with RNA reads differing by more than 2-fold between the WT and *Δefp* strains, the transcript sequence was analyzed using ARNold to identify intrinsic terminators (40–42, 53). From a total of 255 genes, 49 were predicted to contain an intrinsic terminator. Of these 49 genes, only 15 had the predicted intrinsic terminator within 500 bp downstream of a PPX motif (see Table S1 in the supplemental material). Five hundred base pairs was selected as an arbitrary cutoff, since transcription and translation are tightly coupled. Two potential terminators, *narY* and *rsxC*, were chosen for further analysis.

**FIG 6:**
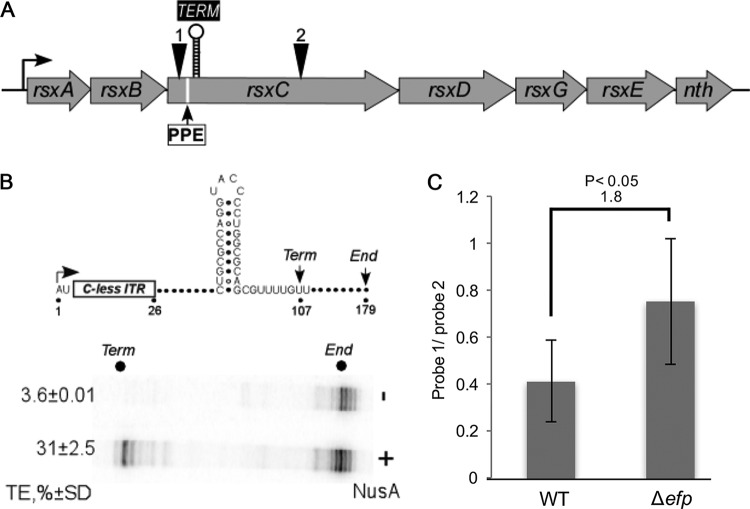
*In vivo* probing for *rsxC*. (A) The *rsxABCDGE-nth* operon (regulonDB) with representation of the location of the polyproline motif, predicted terminator, and the probes for R1 (1) and R2 (2). (B) Termination assay to test the functionality of the predicted intrinsic terminator (ITR) for *rsxC*. NusA, which is essential for termination at some sites, was added to 500 nM where indicated. The means for at least three biological replicates are shown, and error bars indicate 1 standard deviation. (C) Quantification of dot blot probed with probes R1 and R2 for WT and *Δefp* strains. The means for at least three biological replicates are shown, and error bars indicate 1 standard deviation.

**FIG 7:**
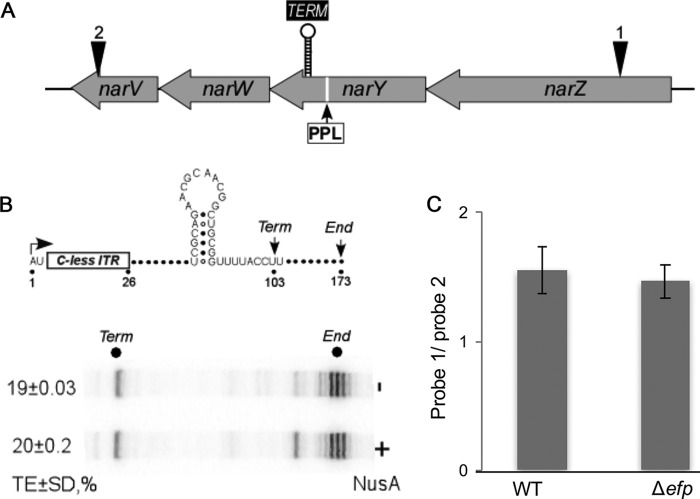
*In vivo* probing for *narY*. (A) The *narZYWV* operon (regulonDB) with representation of the polyproline motif, predicted terminator and the site of the probe for narZ (1) and narV (2). (B) Termination assay to test the functionality of the predicted intrinsic terminator for *narY*. NusA, which is essential for termination at some sites, was added to 500 nM where indicated. The mean for at least three biological replicates is shown and error bars indicate one standard deviation. TE, termination. (C) Quantification of dot blot probed for *narZ* and *narV* for WT and *Δefp* strains. The means for at least three biological replicates are shown, and error bars indicate 1 standard deviation.

To test whether the signals in *narY* and *rsxC* induced termination *in vitro*, as would be expected from an intrinsic terminator, we cloned them downstream from a strong λP_R_ promoter (Fig. 6A and 7A) and carried out single-round elongation assays with *E. coli* RNAP. We found that the *rsxC* signal induced termination only in the presence of NusA (Fig. 6B), an abundant and essential transcription elongation factor that increases pausing and termination at a subset of cellular signals in *E. coli* and other bacteria (54). The terminator in *rsxC* is only 16 nt downstream of a PPE motif (Fig. 6A). *In vivo* probing of *rsxC* mRNA with probes complementary to sequences before and after the PPX motif detected a 1.8-fold reduction of longer transcript in the *Δefp* strain compared to the WT (Fig. 6C; see Fig. S2B in the supplemental material). For *narY*, while the predicted terminator functioned *in vitro* (Fig. 7B), no significant difference was detected between the WT and *Δefp* strains *in vivo* (Fig. 7C and Fig. S2C). The *narY* hairpin is 323 nt downstream of the PPX motif, and this increased spacing could explain the lack of an EF-P-dependent effect if coupling is interrupted by a different mechanism in the intervening region.

## DISCUSSION

In this study, we show that an EF-P-dependent motif upstream of a *rut* site or an intrinsic terminator can decrease expression of a downstream gene in the absence of EF-P. This indicates that by stalling at polyproline motifs, ribosomes in *Δefp* cells can uncouple transcription from translation, thereby unmasking downstream transcription termination signals. Taken together, these data reveal that, in addition to its well-documented role in translation, EF-P can affect gene expression at the level of transcription. Transcription termination is a common regulatory checkpoint in bacteria with a range of molecular mechanisms modulating the termination activity of RNAP. RNAP responds to two different termination signals: Rho-dependent terminators or hairpin-dependent intrinsic terminators (50). Rho utilization (*rut*) sites are characterized by high-C/low-G content, and relatively little secondary structure (9, 33). In this work, we utilized an artificial *rut* site composed of 15 recombinant U recombinant C (rUrC) repeats. This simple polypyrimidine repeat was reported by Guérin et al. to act as an efficient Rho-dependent terminator *in vivo* and *in vitro* (32). They observed termination only in the absence of protein synthesis, when the RNA was available for Rho recruitment. Here, we detected Rho-dependent transcription termination only in *Δefp* cells that included a polyproline motif upstream of the TC repeats. This indicated that the translational stall caused by a polyproline motif was sufficient to trigger uncoupling from transcription and that EF-P restored coupling (Fig. 2). Similar effects of EF-P on coupling were found with the intrinsic terminator *pyrL.* Regulation of the pyrimidine biosynthetic operon (*pyrBI*) is an example of transcription attenuation in *E. coli* (4) that utilizes coupling of translation to transcription of the leader peptide encoded by *pyrL*. Tight coupling across the leader peptide is necessary to prevent formation of the terminator (4). With our reporter, we detected a significant increase in termination efficiency in Δ*efp* cells that included a polyproline motif upstream of *pyrL* compared to a proline-free motif. This increase in termination efficiency was not detected in WT cells (Fig. 4). Transcription and translation are coupled in both *Bacteria* and *Archaea*, raising the possibility that the archaeal EF-P homolog, aIF5A, may play a similar role. Although coupling is absent in *Eukarya*, it is possible that eIF5A has a role in the no-go decay (NGD) mechanism, which recognizes mRNAs with translation elongation stalls and targets them for endonucleolytic cleavage (12).

A closely coupled ribosome protects the elongating RNAP from a premature attack by Rho and from falling into an arrested state. Accelerating the RNAP or slowing down the ribosome would lead to the loss of coupling, which potentially compromises mRNA and genome integrity (6). While the rate of RNA chain synthesis can be modulated by diverse inputs, it appears contrived that the only cellular factors that increase this rate, those from the universally conserved NusG family, also physically link the RNAP and the ribosome (55), forcing them to move in sync. Similarly, factors that safeguard coupling between transcription and translation by accelerating the ribosome are expected to exist in prokaryotes. Our results implicate a universally conserved EF-P as one of these factors, with others to be discovered.

While we argue that EF-P plays an important role in decreasing transcription termination in translated regions, it is conceivable that EF-P may have an opposite effect in regulatory regions. Many control mechanisms than can either increase or decrease termination efficiency have been reported (50). Ribosomes stalling at polyproline motifs may favor formation of a terminator or antiterminator RNA structure, thus enhancing or decreasing termination efficiency, respectively. While ribosome stalling typically leads to increased Rho-dependent termination, the opposite occurs in the *E. coli tna* operon where the stalled ribosome occupies the overlapping *rut* sequences, preventing Rho binding and increasing transcription of the *tna* operon (56). Similarly, in the *Salmonella mgtA* riboswitch, the ribosome stalls during translation of a short proline-rich leader when proline levels are limiting (*mgtL*). This stall favors the formation of a stem-loop that masks the region where Rho interacts. In contrast, complete translation of *mgtL* would promote Rho-dependent transcription termination by exposing this region (57). Interestingly, *rut* sites and PPX-encoding sequences share in common C richness. Since the amino acid proline is coded by CCN, having a PPX guarantees at least 50% C. Furthermore, C-rich mRNA regions as short as 8 nt have been identified as *rut* sites (57, 58). Thus, it would not be surprising for a *rut* site itself to include a PPX, which would protect it from being accessed by Rho during translation (59, 60). Furthermore, Nam et al. have recently shown that EF-P is not constitutively expressed in *Salmonella enterica* serovar Typhimurium (61). The intracellular pathogen decreases *efp* mRNA levels during the course of infection, which selectively stimulates expression of the virulence *mgtC* gene by inducing ribosome stalling at the consecutive proline codons of the *mgtP* open reading frame in the *mgtCBR* leader RNA, thus allowing the formation of a stem-loop structure promoting transcription of the *mgtC* gene (61).

Overall, our findings reveal that the presence of EF-P during translation of polyproline motifs plays a dual role; the first is to support a proper translation elongation rate across this mRNA region, and the second is to maintain coupling between ribosomes and RNAP when potential terminators are transcribed downstream (Fig. 1). Specifically, we found that EF-P maintained coupling of translation and transcription in mRNAs with a *rut* site or an intrinsic terminator downstream of a polyproline motif. Extensive experimentation guided by bioinformatics is now necessary to identify the degree to which potentially EF-P-altered transcription termination sites contribute globally to gene expression.

## MATERIALS AND METHODS

### Strains and plasmids.

The wild-type *E. coli* strain (BW25113) and the Δ*efp* mutant were from the Keio knockout collection, and kanamycin cassettes were removed using FLP recombinase (62, 63). The sequences of the oligonucleotides used in this study are shown in Table S2 in the supplemental material. pBAD30XS (22, 23) was modified by replacing *gfp* with *sfgfp* while maintaining the XhoI-SpeI cloning site. *mCherry* was modified by introducing an i-tag upstream (64). *sfgfp* was N-terminally tagged with (HA)_3_ (HA stands for hemagglutinin), generating pBAD30HA (Fig. S4). For the *rut* reporter pBAD30HATC (Fig. S5), the annealed product of oligonucleotide STC1 and STC2 was inserted in pBAD30HA at the SpeI site in *sfgfp.* This introduced a linker (SGSGSGSG) followed by the *rut* site, (TC)_15_. pBAD30HATC was further double digested with XhoI and SpeI to insert either PPPPPP or PPG by annealed oligonucleotide cloning with oligonucleotide pairs PG1 and PG2 or 6P1 and 6P2, respectively. For the hairpin reporter, a PCR product amplifying only itag-*mCherry* from pBAD30HA with a forward primer introducing SpeI-Pcil (SPT1) and a reverse primer with XbaI (SPT2) was ligated back into pBAD30HA (double digested with SpeI and XbaI) to make pBAD30HAT. The *pyrBI* operon leader peptide was amplified from *E. coli* with a forward primer introducing SpeI (PFS) and a reverse primer introducing Pcil (PRP). This was inserted in frame into pBAD30HAT cut with SpeI and Pcil. This resulting plasmid, pBAD30HAPYT, was further double digested with XhoI and SpeI to insert PPPPPP by annealed oligonucleotide cloning with oligonucleotide pair (6P1 and 6P2) to produce pBAD30HAPYT2. For Psu production from pIA280PSU, a synthetic cassette (gene block from IDT) was cloned between BglII and HindIII in pET28a, replacing T7P with Ptrc and Psu. A mutant Psu, E56K mutant Psu (unable to bind Rho or inhibit Rho-dependent termination [38]) was also cloned (pIA280PSU56). The putative intrinsic terminators were cloned downstream of the bacteriophage λPR promoter. Synthetic oligonucleotide cassettes (2390 plus 2391; RsxC and 2394 plus 2395; NarY) were ligated into SpeI and BglII sites of pIA226 (65). Templates for *in vitro* transcription assays were generated by PCR with oligonucleotides 17 and 1832. *E. coli* RNA polymerase (66) and NusA (67) were purified as described previously. For the plasmid for intrinsic terminator pIA1239, the λP_R_-A26-RsxC putative intrinsic terminator and oligonucleotide cassette 2390 plus 2391 were cloned between the SpeI and BglII sites of pIA226 (pMB1 origin; Amp^r^). For the plasmid for intrinsic terminator pIA1241, the λP_R_-A26-NarY putative intrinsic terminator and oligonucleotide cassette 2394 plus 2395 were cloned between the SpeI and BglII sites of pIA226 (pMB1 origin; Amp^r^)***.***

### Fluorescence assay in *E. coli.*

As described previously, overnight cultures of *E. coli* harboring the constructs were used to inoculate M9 medium to an optical density at 600 nm (OD_600_) of 0.05 (22, 23). The M9 medium was supplemented with 0.2% glycerol, 0.5 g/liter tryptone, 0.3 g/liter thiamine, 0.2% arabinose, 50 µg/ml kanamycin, and 100 µg/ml ampicillin. Cultures were grown at 37°C with shaking. After 2 h, cultures were induced with 0.2 mM isopropyl-β-d-thiogalactopyranoside (IPTG). Fluorescence was measured 5 h after induction using a spectrofluorimeter (Horiba) with excitation at 481 nm and emission at 507 nm for GFP or excitation at 587 nm and emission at 610 nm for mCherry. Fluorescence of strains with an empty vector(s) was subtracted as background from the fluorescence reads. Comparisons of fluorescence between different reporters within the same strain background are indicated on the graphs.

### Termination assays.

Halted transcription elongation complexes were formed for 15 min at 37°C with 30 nM linear PCR-generated template (pIA1239 or pIA1241) and 40 nM RNAP holoenzyme in 50 µl of 20 mM Tris-acetate, 20 mM Na-acetate, 2 mM Mg-acetate, 14 mM 2-mercaptoethanol, 0.1 mM EDTA, 4% glycerol, pH 7.9. To halt RNAP after the addition of A26, synthesis was initiated in the absence of CTP, with 150 µM ApU, 5 µM ATP and UTP, and 1 µM GTP supplemented with 10 µCi of [α-^32^P]CTP (3,000 Ci/mmol). Halted complexes were incubated with 500 nM NusA (or storage buffer) for 3 min at 37°C. Transcription was restarted by addition of 1/10 volume of 0.5 mM nucleoside triphosphates (NTPs) and 100 µg ml^−1^ heparin. Following 10-min incubation at 37°C, samples were quenched by addition of an equal volume of 10 M urea, 50 mM EDTA, 45 mM Tris-borate (pH 8.3), 0.1% bromophenol blue, and 0.1% xylene cyanol. RNAs were separated on 8% denaturing polyacrylamide gels and quantified using a Typhoon FLA 9000 scanner (GE Healthcare), ImageQuant software, and Microsoft Excel. Each assay was performed in triplicate.

### *In vivo* probing of potential terminators.

For *cadA* probing, the WT *E. coli* and *Δefp* mutant strain harboring pIA280PSU or pIA280PSUEK were grown in LB adjusted to pH 5.8 with 100 mM sodium phosphate buffer (68). At an OD_600_ of 0.5, cultures were induced with 0.1 mM IPTG. Ninety minutes postinduction, the cells were harvested to isolate RNA. For *hslU* and *ygdH* probing, the procedure was described above but with no pH adjustment for LB. For *narY* and *rsxC* probing, the WT *E. coli* and *Δefp* strain were grown in LB. At an OD_600_ of 1.5, cells were harvested to isolate RNA.

### Isolation and analyses of *in vivo* RNA transcripts.

After fluorescence measurements, cultures were pelleted and stored in RNAlater stabilization solution (Ambion) and kept at 4°C. Total bacterial RNA was extracted as follows. The RNAlater stabilization solution was removed, and the pellet was resuspended in REB buffer and extracted as described previously (69). Northern blot analysis was performed by fractionating 20 µg of total RNA on a 1.5% agarose gel containing 6% formaldehyde (70–72). The RNA was transferred onto Zeta-probe cationized nylon membrane (Bio-Rad) by capillary gel transfer (71). RNA was then UV cross-linked to the membrane followed by prehybridization for 2 to 4 h at 50°C in a solution containing 5× SSC (1× SSC is 0.15 M NaCl plus 0.015 M sodium citrate), 20 mM Na_2_HPO_4_ (pH 7.2), 1× Denhardt solution, 7% SDS, and 100 µg/ml denatured salmon sperm DNA. After addition of the denatured 5′-end ^32^P-labeled DNA probe, the hybridization was allowed to proceed for 18 h at 50°C. The membrane was washed at 50°C for 30 min: twice in 3× SSC, 10× Denhardt solution, 25 mM NaH_2_PO_4_ (pH 7.5), and 5% SDS and once in 1% SSC and 1% SDS. For dot blots, the RNA was loaded to the nylon membrane using the Bio-Dot microfiltration apparatus as described previously (73). The membrane was subsequently processed as described above.

### Statistical analysis.

All *P* values were determined by unpaired Student’s *t* test.

## SUPPLEMENTAL MATERIAL

Figure S1  (A) GFP fluorescence/OD for the samples in Fig. 2B. WT and *Δefp* strains harbored the *rut* reporter and a compatible plasmid producing either active or inactive E56K mutant Psu. The values shown on a gray background are the fold changes in GFP fluorescence with coexpression of Psu. The means for at least three biological replicates are shown, and error bars indicate 1 standard deviation. (B) Representative dot blot for the *rpoB2* samples in Fig. 3. The values for the mCherry probe were normalized against those for the HA probe. The ratio of signal for active/inactive Psu is shown. The means for at least three biological replicates are shown, and error bars indicate 1 standard deviation. (C) Representative dot blot for *mCherry/pyrL-mCherry* for the samples in Fig. 4B. The values for the mCherry probe were normalized against those for the HA probe. The ratio of GN/P6 signal with hairpin (+) and without a hairpin (−) is shown. The means for at least three biological replicates are shown, and error bars indicate 1 standard deviation. Download Figure S1, PDF file, 0.3 MB

Figure S2  *In vivo* probing by dot blotting. (A) Dot blot for WT and *Δefp* strains with and without Psu probed with the C1 and C2 probes. (B) Dot blot for WT and *Δefp* strains probed with *rsxC* probes R1 and R2. (C) Dot blot for WT and *Δefp* strains probed for *narZ* and *narV*. Download Figure S2, PDF file, 0.2 MB

Figure S3  Quantification for dot blot of WT and *Δefp* strains with and without Psu probed with hslU1 and hslU2 probe (dark gray bars) and similar analysis for the samples probed with ydgH1 and ydgH2 (light gray bars). The means for at least three biological replicates are shown, and error bars indicate one standard deviation. Download Figure S3, PDF file, 0.2 MB

Figure S4  Map of pBAD30HA. Download Figure S4, PDF file, 0.1 MB

Figure S5  Map of pBAD30HATC. Download Figure S5, PDF file, 0.1 MB

Table S1  Prediction of intrinsic terminators for genes with RNA reads more than 2-fold different in WT and *Δefp* strains from RNA-seq data in reference 23 Table S1, PDF file, 0.1 MB

Table S2  Oligonucleotide probes utilized in this work. Table S2, PDF file, 0.1 MB
