# Supplementary material for: Maintenance of Transcription-Translation Coupling by Elongation Factor P
Source: mBio. 2016 Sep 13;7(5):e01373-16. doi: 10.1128/mBio.01373-16 (PMC5021804; doi:10.1128/mBio.01373-16)
Supplement: Figure S1 — (A) GFP fluorescence/OD for the samples in Fig. 2B. WT and Δefp strains harbored the rut reporter and a compatible plasmid producing either active or inactive E56K mutant Psu. The values shown on a gray background are the fold changes in GFP fluorescence with coexpression of Psu. The means for at least three biological replicates are shown, and error bars indicate 1 standard deviation. (B) Representative dot blot for the rpoB2 samples in Fig. 3. The values for the mCherry probe were normalized against those for the HA probe. The ratio of signal for active/inactive Psu is shown. The means for at least three biological replicates are shown, and error bars indicate 1 standard deviation. (C) Representative dot blot for mCherry/pyrL-mCherry for the samples in Fig. 4B. The values for the mCherry probe were normalized against those for the HA probe. The ratio of GN/P6 signal with hairpin (+) and without a hairpin (−) is shown. The means for at least three biological replicates are shown, and error bars indicate 1 standard deviation. Download [file mbo004162983sf1.pdf]

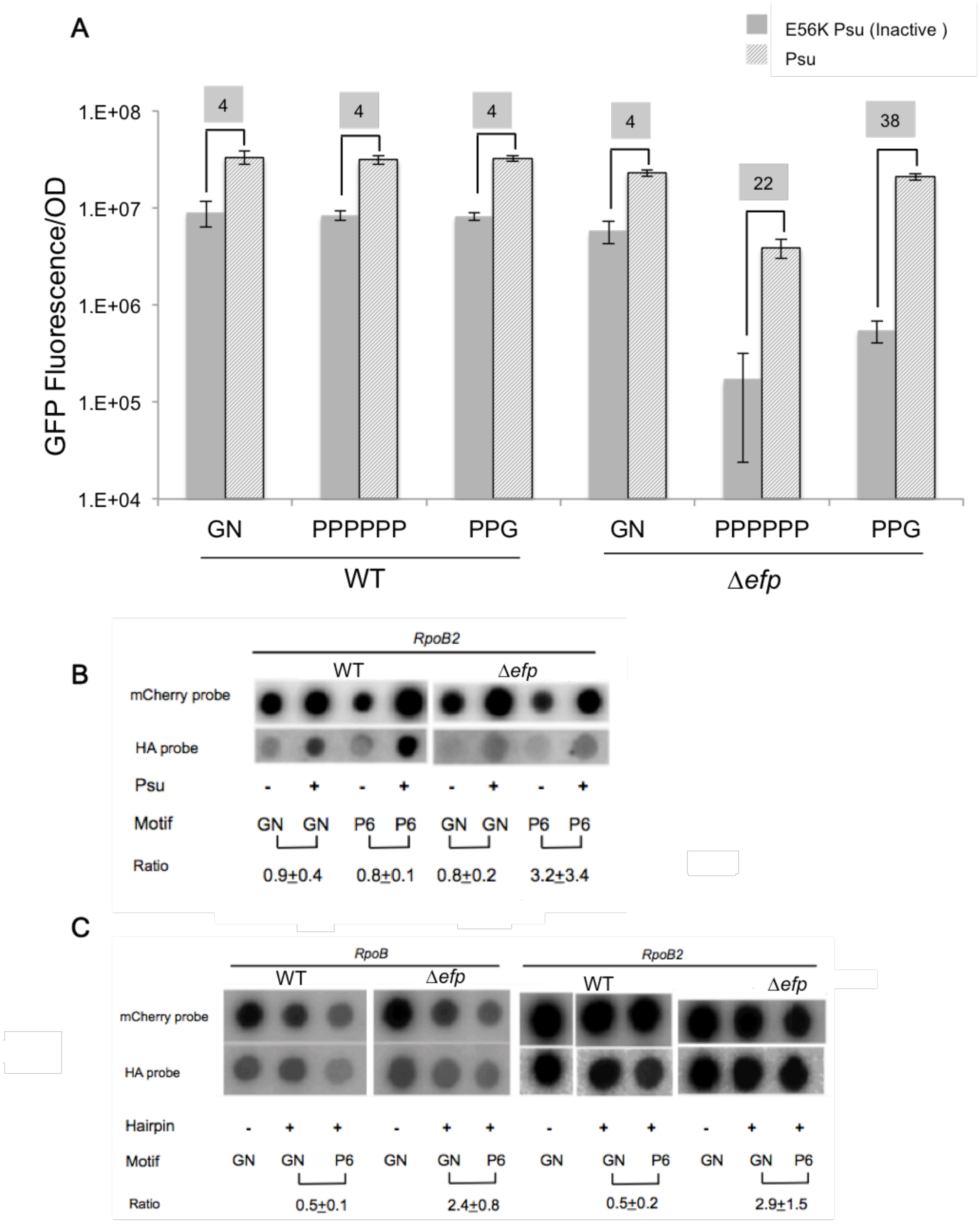

Figure S1. A) GFP fluorescence/OD for the samples in fig. 2B. WT and Δ*efp* strains harbored the *rut* reporter and a compatible plasmid producing either active or inactive E56K mutant Psu. The values highlighted in grey are the fold change in GFP fluorescence with co-expression of Psu. The mean for at least three biological replicates is shown and error bars indicate one

standard deviation. B) Representative dot blot for the *rpoB2* samples in fig. 3, values from mCherry probe were normalized against HA probe. The ratio of signal for active/inactive Psu is shown. The mean for at least three biological replicates is shown and error bars indicate one standard deviation. C) Representative dot blot for *mCherry/pyrL-mCherry* for the samples in fig. 4B, values from mCherry probe were normalized against HA probe. The ratio of GN/P6 signal for hairpin + patterns is shown. The mean for at least three biological replicates is shown and error bars indicate one standard deviation.
