# Supplementary material for: Maintenance of Transcription-Translation Coupling by Elongation Factor P
Source: mBio. 2016 Sep 13;7(5):e01373-16. doi: 10.1128/mBio.01373-16 (PMC5021804; doi:10.1128/mBio.01373-16)
Supplement: Figure S2 — In vivo probing by dot blotting. (A) Dot blot for WT and Δefp strains with and without Psu probed with the C1 and C2 probes. (B) Dot blot for WT and Δefp strains probed with rsxC probes R1 and R2. (C) Dot blot for WT and Δefp strains probed for narZ and narV. Download [file mbo004162983sf2.pdf]

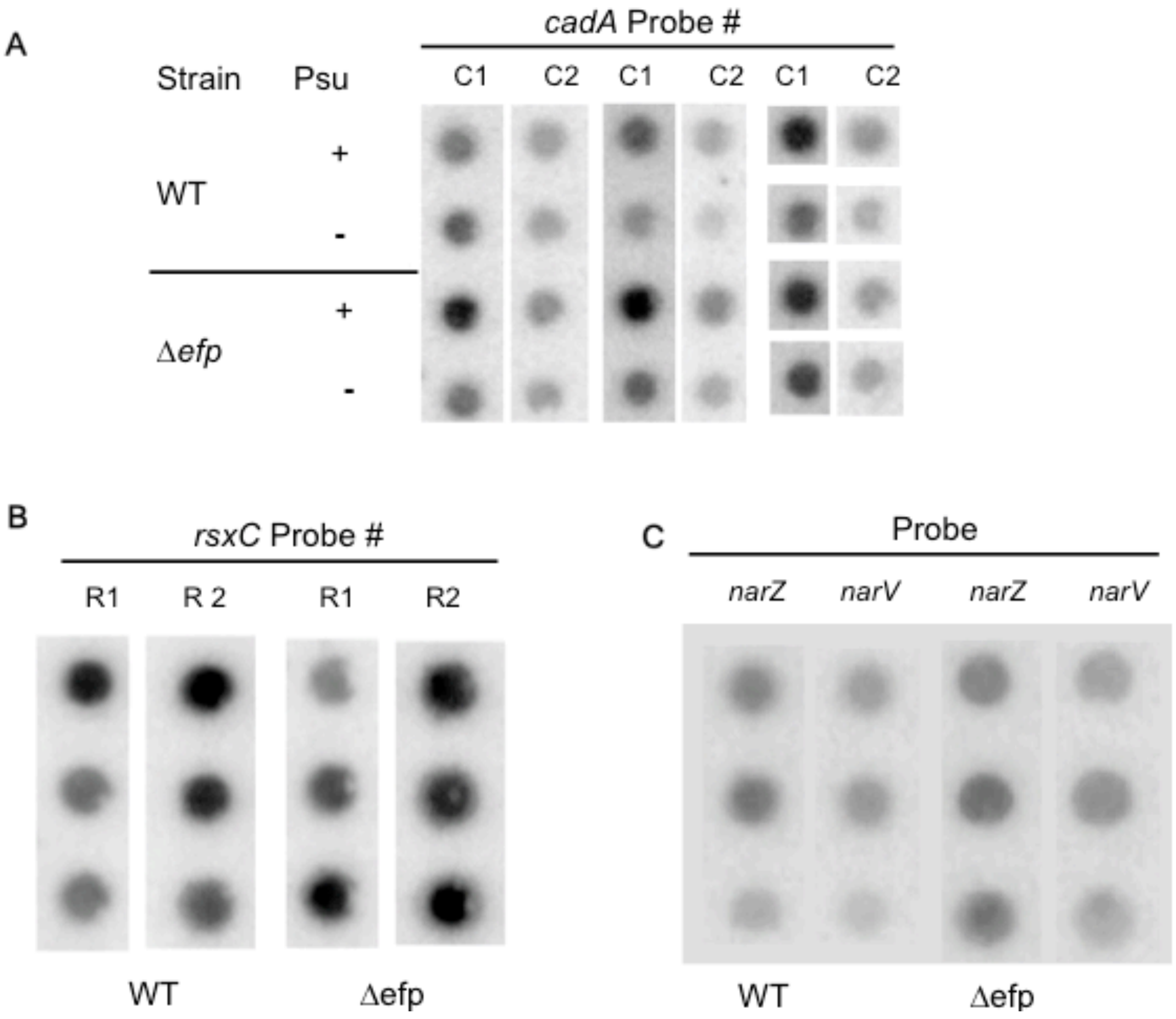

Figure S2. *In vivo* probing by dot blot. A) Dot blot for Wt and Δ*efp* with and without Psu probed with C1 and C2 probe. B) Dot blot for Wt and Δ*efp* probed with rsxC probe R1 and R2. C) Dot blot probed for *narZ* and *narV* for WT and Δ*efp.*
