# Supplementary material for: Maintenance of Transcription-Translation Coupling by Elongation Factor P
Source: mBio. 2016 Sep 13;7(5):e01373-16. doi: 10.1128/mBio.01373-16 (PMC5021804; doi:10.1128/mBio.01373-16)
Supplement: Figure S3 — Quantification for dot blot of WT and Δefp strains with and without Psu probed with hslU1 and hslU2 probe (dark gray bars) and similar analysis for the samples probed with ydgH1 and ydgH2 (light gray bars). The means for at least three biological replicates are shown, and error bars indicate one standard deviation. Download [file mbo004162983sf3.pdf]

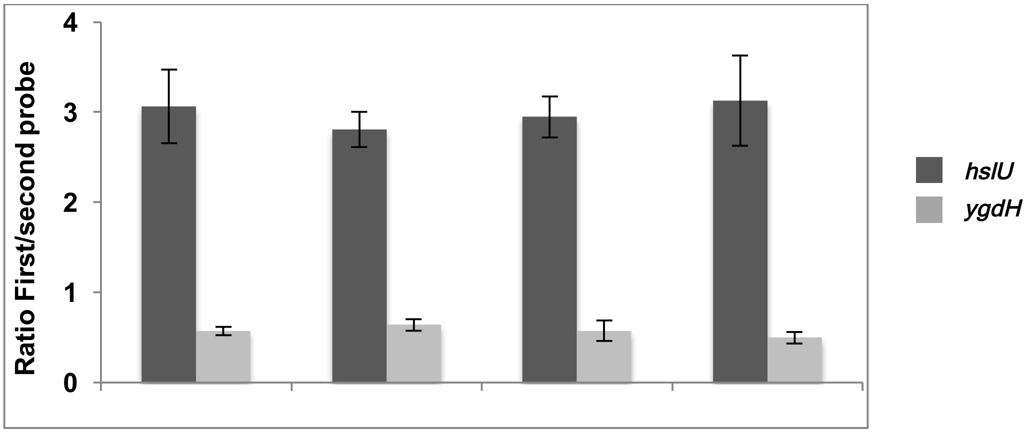

Strain	WT		Δ*efp*	
Psu	+	-	+	-

Figure S3. Quantification for dot blot of Wt and Δ*efp* with and without Psu probed with hslU1 and hslU2 probe (dark grey bars) and similar analysis for the samples probed with ydgH1 and ydgH2 (light grey bars). The mean for at least three biological replicates is shown and error bars indicate one standard deviation.
