# Supplementary figures and images for: Maintenance of Transcription-Translation Coupling by Elongation Factor P
Source: mBio. 2016 Sep 13;7(5):e01373-16. doi: 10.1128/mBio.01373-16 (PMC5021804; doi:10.1128/mBio.01373-16)

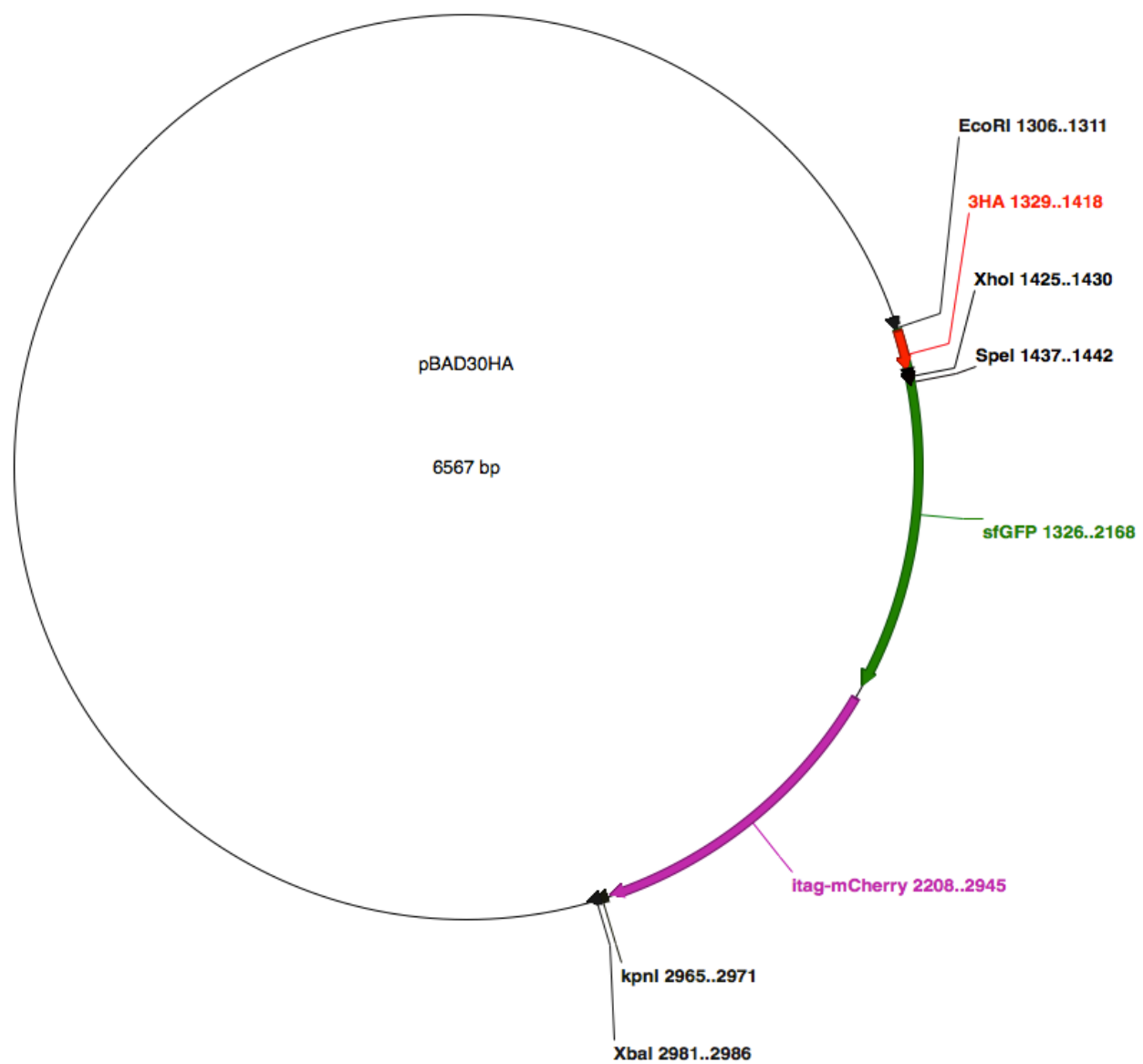

**Figure S4.** Map of pBADHA

Supplement: Figure S4 — Map of pBAD30HA. Download [file mbo004162983sf4.pdf]

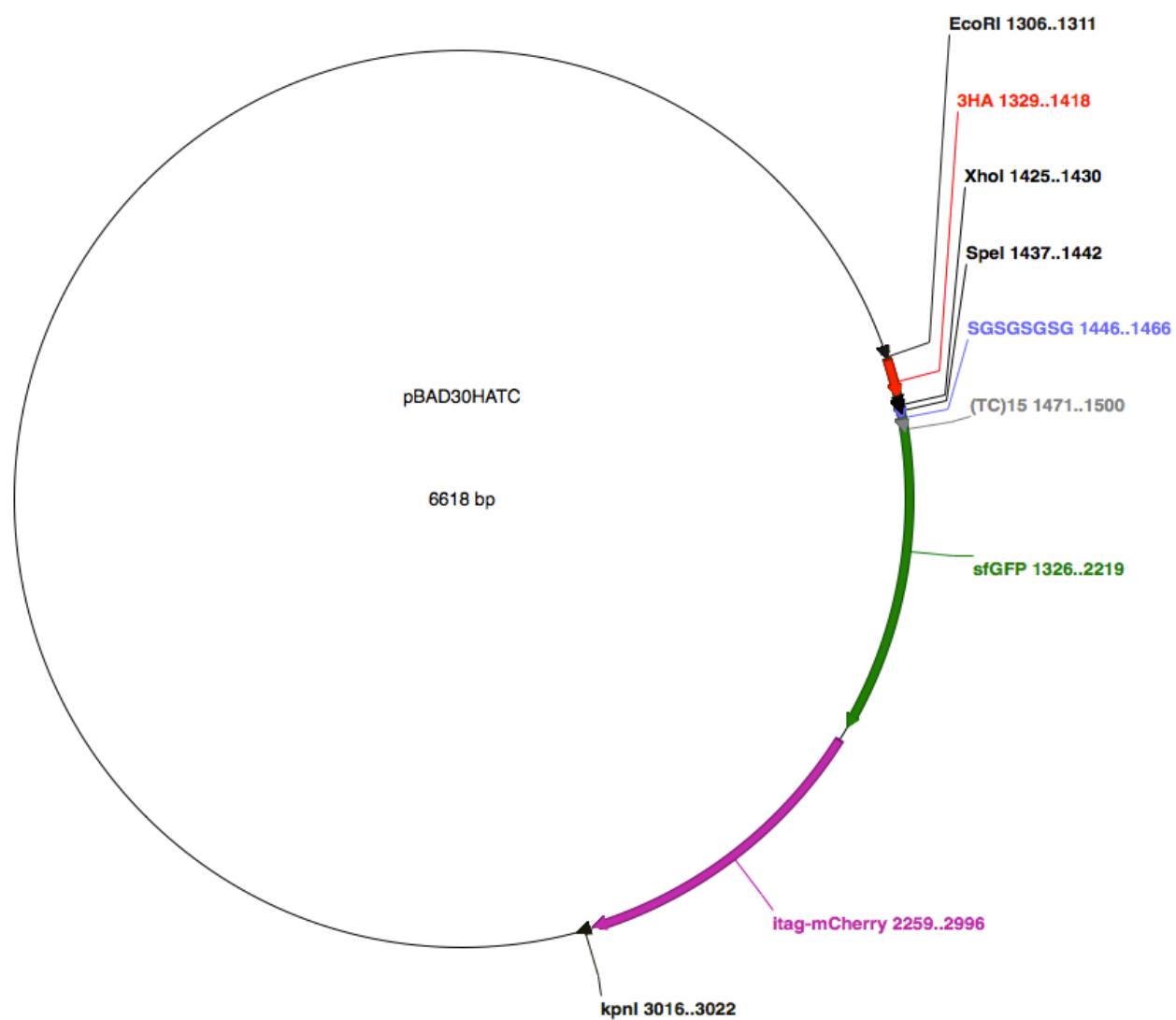

**Figure S5.** Map of pBADHATC

Supplement: Figure S5 — Map of pBAD30HATC. Download [file mbo004162983sf5.pdf]
