# Supplementary material for: Maintenance of Transcription-Translation Coupling by Elongation Factor P
Source: mBio. 2016 Sep 13;7(5):e01373-16. doi: 10.1128/mBio.01373-16 (PMC5021804; doi:10.1128/mBio.01373-16)
Supplement: Table S1 — Prediction of intrinsic terminators for genes with RNA reads more than 2-fold different in WT and Δefp strains from RNA-seq data in reference 23 [file mbo004162983st1.pdf]

Table S1: Prediction of intrinsic terminators for genes with RNA reads more than 2 fold different between in WT versus Δ*efp* from RNA-seq data in (1)

Gene *	5' TTS end	Detected Secondary structures and free energy of stem-loop region (kcal/mol) **		PPX	bps between TTS & PPX ***
yahG	211	GAGCGTATTATCCAGTCGCATCCGGTGCTGATTGGTTTTG ATCAGGC	-5.6	PPG	90
citT	473	CCAACACCGCGCGTACCGGGGGTACGgTTTTTCCGGTCA	-7.2	PPQ	373
xanP	107	TGTTTGCCGCCTGTCAGCATCTGCTGGCGaTGTTCGTTGC GG	-9.6	PPL	10
brnQ	263	TTTGTTACCTGGCGGTGGGGCCGCTTTTCGCTACGC	-6.6	PPM	166
entS	1125	ACCGGTTGCTTCCGCAAGCGCGAGCGGTTTTGGTTTGTT	-5.7	PPPP	514
proY	1209	AACGACCATCGGCGGGCTGATTTTCCTGCTCTTTATTATC G	-6.3	PPE	41
psuT	253	AAAATGGATGTCCTGTTTGACGGTGCGGGTTTTATCTTC GC	-5.2	PPG	96
yhhJ	770	CGCTGTTTATGCTGGGCGTGGCGCTCAGTCTGTTTGCCAC	-8.7	PPN	457
ymfE	573	TTTTGTAATTTTATCATTCTCAGTGAGTGGTATTATCTT TGCTT	-7.4	PPI	416
clcB	1130	TATGTGAAATGACCGGGGAGTATCAGCTACTCCCCGGTT TATTGATTGCCT	-14.8	PPL	247
flhC	396	GCAACTTTCCAGCTGCAACTGCTGCGGCGGCaaTTTTATT ACCCA	-7.1	PPK	233
narY	1398	GGGCGATGCCTTCGCAGAACGCAACGGCTGCGGTTTTACC TTTGG	-6.6	PPL	323
nrdD	1781	AAGCGCCTTACCCGCCGCTGGCGAACGGTGGTTTCATTTG CTA	-9.3	PPL	Within
rsxC	89	ACGGTACACCCCTGCGCCAGGTACCCCTGGCGCAGcgTTT TGTTATTCC	-15.1	PPE	16
yhjA	534	AAAACTGCCTACCGATGCGCAAAAAGTGGCGTTGGgTTT TGCGCTGTA	-9.1	PPL	208

*   Genes that have a predicted intrinsic terminator within 500 bps downstream of a PPX
** Prediction of intrinsic terminators done with ARNold (2-5). Secondary structures colored such that loops are in red and stems in blue. Lowercase letters in RNAmotif predictions indicate the spacer element, between the stem-loop and T-rich region.
*** This column shows the # of nts between the predicted transcription terminator site (TTS) and the closest PPX
