# Supplementary material for: Maintenance of Transcription-Translation Coupling by Elongation Factor P
Source: mBio. 2016 Sep 13;7(5):e01373-16. doi: 10.1128/mBio.01373-16 (PMC5021804; doi:10.1128/mBio.01373-16)
Supplement: Table S2 — Oligonucleotide probes utilized in this work. [file mbo004162983st2.pdf]

Table S2: Oligonucleotide probes utilized in this work.

Name	Sequence (5'-3')
STC1	CTAGTGGCAGCGGCAGCGGCAGCGGCAGCGACGTCTCTCTCTCT CTCTCTCTCTCTCTCTCTCGGCCGG
STC2	CTAGCCGGCCGAGAGAGAGAGAGAGAGAGAGAGAGAGAGAGACG TCGCTGCCGCTGCCGCTGCCGCTGCCA
PG1	TCGAGCCGCCGGGTA
PG2	CTAGTACCCGGCGGC
6P1	TCGAGCCGCCGCCGCCGCCGCCGA
6P2	CTAGTCGGCGGCGGCGGCGGCGGC
SPT1	CCTAACACTAGTGGCAACACATGTCCGACATTAGAAATAGCAC AAAAAAAAGTGAGC
SPT2	CAGAATTTGCCTGGCGGCAGTA
PFS	GGCAACACTAGTATGGTTCAGTGTGTTCGACATTTTGTC
PRP	ATGTCGGACATGTTCTCCTGACGCCTGGGCAAAAAAAAGC
mCherry probe	TGTTATCTTCTTCGCCCTTGCTCACTTTTTTTTGTGCTATTTCTAA TGTC
HA probe	GTTGCCCTCGAGTTTGCTAGCGTAATCTGGAACGTCATATGGAT AGG
5S probe (ppB10, (6))	ACACTACCATCGGCGCTACG
C1	ATCATATTGGCGTTAATACGACCTACCATTTCGTCGAGG
C2	TTATTTTTTGCTTTCTTCTTTCAATACCTTAACGGTATA
R1	CCGGTGGATGGATGCCGCCGTTGAAATCCCAGATTTTATTTTTT CTGAAT
R2	CGTTCGCCCGCTTTAATCACAATAGGCTGTGTAGCCTGGGCCTG TTTCTC
narV	CGCACAATTTGATATCGACGAGTAAAGTACTCAAACGGCGCGC TC
narZ	CAGGTCGCAATACCAGTCGTAGAAACTTAAACAGGTGCCGCCA AG
hslU1	GTCAGATCGCGAATAATAGAATCCACTTCCTTACCGACGTA
hslU2	CACATAATCTGCGTCAATAGTGATATTTTGACCGCTTAAATCGC
ygdH1	CGGCGCAAGACGTTAATATCGAAATTTTCAAAACGAG
ygdH2	CGACGCATAATCTCTTTATCGCCGTTGATTTTGTAAGGACC
17	CGTTAAATCTATCACCGCAAGG
1832	TCTCATGCGTTCATGCACCACTGG

2390	CTAGCCCTGCGCCAGGTACCCCTGGCGCAGcgTTTTGTTATTA
2391	GATCTAATAACAAAAcgCTGCGCCAGGGGTACCTGGCGCAGGG
2394	CTAGCTCGCAGAACGCAACGGCTGCGGTTTTACCTTT
2395	GATCAAAGGTAAAACCGCAGCCGTTGCGTTCTGCGAG

1. **Elgamal S, Katz A, Hersch SJ, Newsom D, White P, Navarre WW, Ibba M.** 2014. EF-P dependent pauses integrate proximal and distal signals during translation. PLoS Genet **10:**e1004553.
2. **Gautheret D, Lambert A.** 2001. Direct RNA motif definition and identification from multiple sequence alignments using secondary structure profiles. J Mol Biol **313:**1003-1011.
3. **Hofacker IL, Fontana W, Stadler PF, Bonhoeffer LS, Tacker M, Schuster P.** 1994. Fast folding and comparison of RNA secondary structures. Monatshefte für Chemie / Chemical Monthly **125:**167-188.
4. **Lesnik EA, Sampath R, Levene HB, Henderson TJ, McNeil JA, Ecker DJ.** 2001. Prediction of rho-independent transcriptional terminators in Escherichia coli. Nucleic Acids Res **29:**3583-3594.
5. **Macke TJ, Ecker DJ, Gutell RR, Gautheret D, Case DA, Sampath R.** 2001. RNAMotif, an RNA secondary structure definition and search algorithm. Nucleic Acids Res **29:**4724-4735.
6. **Figueroa-Bossi N, Schwartz A, Guillemardet B, D'Heygere F, Bossi L, Boudvillain M.** 2014. RNA remodeling by bacterial global regulator CsrA promotes Rho-dependent transcription termination. Genes Dev **28:**1239-1251.
